# Effects of obesity and foot arch height on gait mechanics: A cross-sectional study

**DOI:** 10.1371/journal.pone.0260398

**Published:** 2021-11-29

**Authors:** Daekyoo Kim, Cara L. Lewis, Simone V. Gill

**Affiliations:** College of Health and Rehabilitation Science, Sargent College, Boston University, Boston, MA, United States of America; West Virginia University, UNITED STATES

## Abstract

Foot arch structure contributes to lower-limb joint mechanics and gait in adults with obesity. However, it is not well-known if excessive weight and arch height together affect gait mechanics compared to the effects of excessive weight and arch height alone. The purpose of this study was to determine the influences of arch height and obesity on gait mechanics in adults. In this study, 1) dynamic plantar pressure, 2) spatiotemporal gait parameters, 3) foot progression angle, and 4) ankle and knee joint angles and moments were collected in adults with normal weight with normal arch heights (n = 11), normal weight with lower arch heights (n = 10), obesity with normal arch heights (n = 8), and obesity with lower arch heights (n = 18) as they walked at their preferred speed and at a pedestrian standard walking speed, 1.25 m/s. Digital foot pressure data were used to compute a measure of arch height, the Chippaux-Smirak Index (CSI). Our results revealed that BMI and arch height were each associated with particular measures of ankle and knee joint mechanics during walking in healthy young adults: (i) a higher BMI with greater peak internal ankle plantar-flexion moment and (ii) a lower arch height with greater peak internal ankle eversion and abduction moments and peak internal knee abduction moment (i.e., external knee adduction moment). Our results have implications for understanding the role of arch height in reducing musculoskeletal injury risks, improving gait, and increasing physical activity for people living with obesity.

## Introduction

Obesity is a major public health concern worldwide. Obesity increases the risk of other health problems such as heart disease, stroke, type-2 diabetes, osteoarthritis, and certain cancers that may cause premature death [1]. The prevalence of obesity in the United States is 42.4% among adults over 20 years of age and has increased 12% over the past 20 years [2]. To combat obesity, increasing energy expenditure via increasing physical activity level has been strongly recommended; physical activity promotes weight loss, prevents weight gain and regain, and can help maintain cardiovascular and metabolic health [1]. Walking is a common and cost-effective intervention used to increase overall physical activity and to meet the recommended 150 minutes of weekly moderate-to-vigorous physical activity [3]. However, most adults with obesity fall short of these recommendations [4].

Major contributors to decreased physical activity in individuals with obesity include musculoskeletal injury risk due to excessive body weight, specifically in areas that result in greater thigh and trunk girth [5, 6] and waist circumference [7]. Walking requires coordinating motor actions specific to body constraints such as body weight [8]. Compared to adults with normal weight, adults with obese body mass index (BMI) scores show differences in spatiotemporal gait parameters. They take shorter but wider steps by decreasing step length and increasing step width, and walk more slowly [9, 10]. Moreover, obesity affects gait mechanics during walking. Adults with obesity have slower gait velocity, greater absolute ground reaction forces, and altered lower-limb joint loading patterns compared to normal-weight adults [6, 11, 12]. These differences in walking, especially slower gait velocity, are attributed to their attempts to increase stability because of impaired balance [13], to minimize mechanical external work [14], to decrease load at the knee [15], and to curb energy cost and relative effort [16]. However, these differences in walking are actually associated with increasing the risk of musculoskeletal injury [6] and falls in individuals with obesity [17–19].

Adults with obesity tend to have lower arches or “flat feet” based on footprint and plantar pressure measures [20–22]. Feet with lower arches tend to be more flexible during the propulsive phase of walking [23, 24] leading to excessive foot pronation. Individuals with overpronated feet are more likely to have lower-limb malalignment with excessive loads, typically show a greater toe-out angle during walking than normal-weight individuals [10, 25], and consequently develop foot pain such as chronic plantar heel pain [26, 27]. The combination of differences in walking and arch height in adults with obesity contributes to musculoskeletal injuries due to soft tissue damage [25] such as posterior tibial tendon dysfunction [28], ankle sprains [10], and plantar fasciitis [29].

Although spatiotemporal gait, lower-limb joint kinematics and kinetics, and lower-limb malalignment have been shown to differ in adults with obesity compared to adults with normal weight and to contribute to increased musculoskeletal injury risks [15, 30–32], to our knowledge, few studies have directly examined the relationship between gait mechanics and arch height in this population. Our previous studies have examined the center of foot pressure as a proxy for the contribution of arch height to gait [33], but we still have limited information to confirm whether both excessive weight and low arches together result in altered gait kinematics and kinetics compared to the effects of either excessive weight or low arch height alone. The purpose of the present study is to determine the influences of arch height and obesity on gait mechanics in adults with obesity. We hypothesized that BMI and arch height would each be associated with particular measures of ankle and knee joint mechanics during walking in adults at their preferred speed and at a pedestrian standard walking speed, 1.25 m/s: (i) BMI with sagittal plane ankle and knee joint kinematics and kinetics and (ii) arch height with frontal plane ankle and knee joint kinematics and kinetics.

## Materials and methods

### Participants

Forty-seven young adults (25 females) participated in this study from July 2019 through November 2019 (Table 1). Study eligibility included being between 18–35 years old, having no weight loss surgery, having no significant cardiovascular, vestibular, or other neurologic disorders, having no hip, knee, or foot pain on most days during the past 90 days, and having the ability to walk independently on a treadmill for over 40 minutes. All participants gave informed written consent before participating. The Boston University Institutional Review Board approved the protocols.

**Table 1:** Demographics and anthropometric information. Means are listed with standard deviations in parentheses.

	Non-Obese		Obese	
	Normal Arch	Lower Arch	Normal Arch	Lower Arch
	(N = 11)	(N = 10)	(N = 8)	(N = 18)
Age (yrs)	26.38 (6.46)	27.90 (4.24)	28.82 (4.91)	28.37 (3.46)
Height (m)	1.70 (0.07)	1.75 (0.09)	1.69 (0.08)	1.68 (0.08)
Weight (kg)	64.10 (11.92)	71.75 (12.32)	108.79 (22.86)	113.99 (28.69)
BMI (kg/m^2^)	22.15 (2.79)	23.19 (2.20)	37.91 (7.53)	40.05 (8.01)
CSI (%)	33.07 (3.27)	48.65 (2.88)	37.68 (3.72)	59.51 (11.51)
Navicular height (cm)	2.78 (0.34)	2.46 (0.31)	2.04 (0.29)	1.59 (0.22)
Gait Velocity (m/s)	1.24 (0.10)	1.19 (0.05)	1.06 (0.11)	1.04 (0.08)

### Experimental protocols

#### Arch height measures

**Fig 1:**
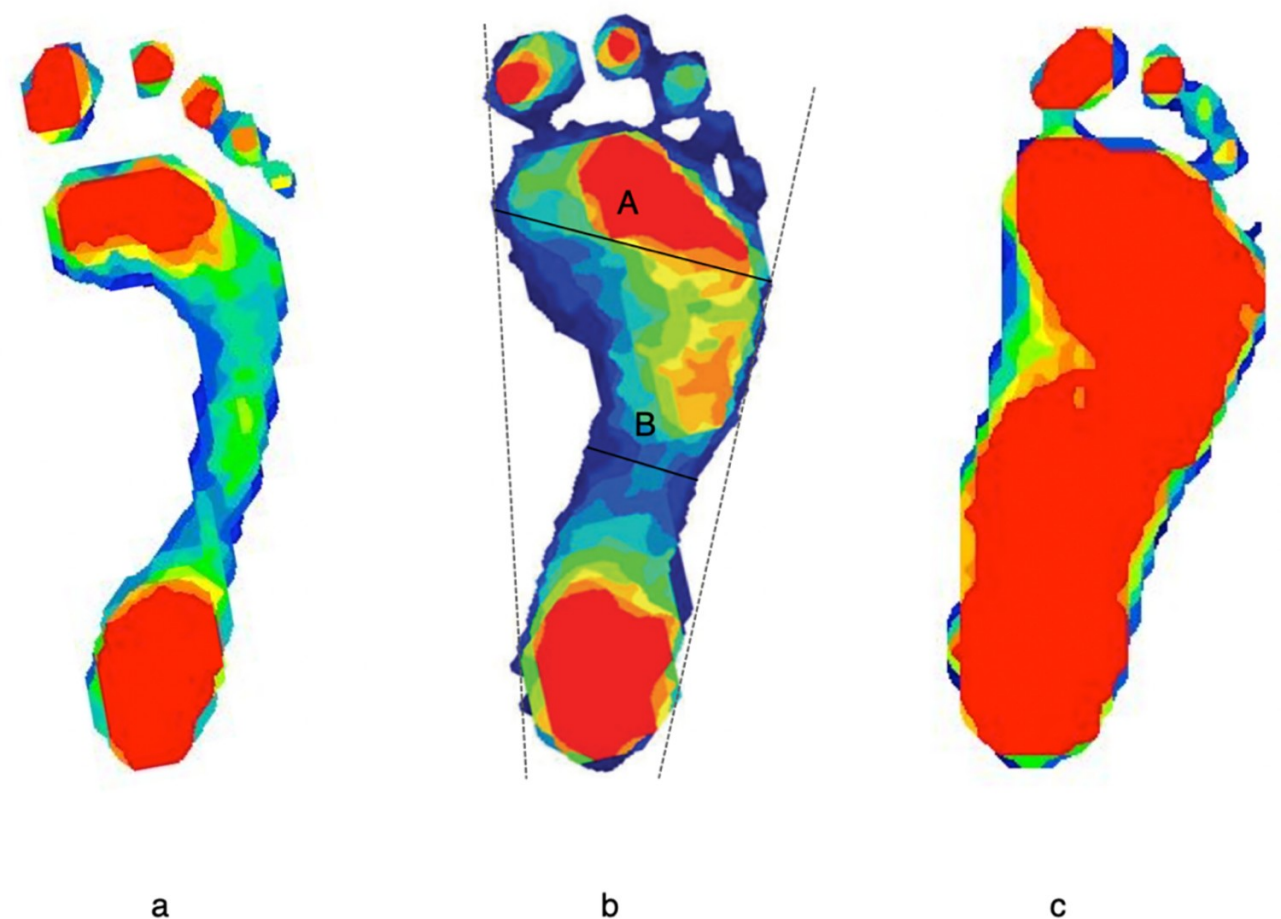
Example of digital foot pressure data. These feet represent three participants: one with a higher arch (a), one with a normal arch (b), and one with a lower arch (c). The colors indicate areas of the feet that exert pressure that is graded from low (blue) to high (red) areas of pressure in kilopascals. The high-arched individual on the left (a) is a 31-year-old male with a BMI of 22.06 kg/*m*^2^. The low-arched individual on the right (c) is male, 28 years old, and has a BMI of 64.14 kg/*m*^2^. The Chippaux-Smirak Index equals the ratio of the smallest distance of the midfoot (B) and the longest distance of the forefoot (A) as described in Fig 1(B).

We estimated participants’ arch height index using i) barefoot plantar pressures and ii) navicular height measures [18]. Dynamic plantar pressure values were recorded with a digital pressure mat after calibrating each participant’s body weight (Tekscan Inc., South Boston, MA). When participants stood barefoot of their dominant leg on the digital pressure mat (488 mm × 447 mm), plantar pressure data were collected via 8,448 sensing elements at the sampling frequency, 185Hz. Tekscan software found the maximum pressure distribution from each sensor, created a peak plantar pressure profile, and imported it to ImageJ for processing (Fig 1). We then measured the midfoot’s smallest width (B) and the largest width of the metatarsal head area (A) as shown in Fig 1B. We finally calculated the Chippaux-Smirak Index (CSI = B/A × 100) [34, 35], which has widely been used to estimate arch height index [18, 36]. Next, we directly measured the navicular height, which is highly associated with the CSI in children and adolescents [37, 38]. We marked the medial side of the head of the navicular tuberosity (for the dominant foot only) with a ballpoint pen from the standing position. We repeatedly measured the distance of the marked position of the navicular tuberosity to the ground using a steel ruler (resolution: 0.5 mm) three times and averaged them.

#### Spatiotemporal gait measures

We used a portable, pressure-sensitive gait carpet (6.10 m long × 0.89 m wide) to measure the distance (x and y coordinates) and timing of each footfall at a spatial resolution of 1.27 cm and a sampling frequency of 120 Hz (Protokinetics, LLC; Peekskill, NY, USA). Step length and width were measured with the absolute difference in anteroposterior and mediolateral center of pressure (COP) position between the right and left foot at heel strike of the right foot (m). Double-limb support time was measured as the period between right foot heel strike and left foot toe-off (s). Participants’ preferred over-ground gait velocity was calculated by dividing total step length by total step time (m/s).

#### Kinematic and kinetic measures

We used a 10-camera motion capture system (Vicon Motion Systems, Oxford, UK; 100 Hz sampling frequency) to capture 44 reflective markers attached to the trunk, pelvis, thighs, shanks and feet. Markers were placed bilaterally on the posterior heel, three metatarsal heads (1^st^, 2^nd^, and 5^th^), medial and lateral malleoli, medial and lateral femoral epicondyles, greater trochanter, anterior superior iliac spine, posterior superior iliac spinae, and acromion process. A single marker was placed on the xiphoid process, jugular notch, 7^th^ cervical vertebra, and 10^th^ thoracic vertebrae. Rigid clusters of four markers were attached to the shank and thigh bilaterally. Raw marker positions were filtered using a second-order low-pass Butterworth filter with a cut-off frequency of 6 Hz. A static standing trial was captured and the positions of markers on segment endpoints were used to calibrate an eight-segment model for each participant using established inertia parameters [39]. Foot progression angle and joint angles for the ankle, knee, and hip were computed in three dimensions as the orientation of the distal segment with reference to the proximal segment and differentiated to calculate joint velocities.

Force data were recorded during walking using the two force plates embedded in an in-ground treadmill (Bertec Corporation, Columbus, OH; 1000 Hz sampling frequency). Raw analog force signals were filtered with a second-order low-pass Butterworth filter with a cut-off frequency of 10 Hz. Lower-limb joint kinematics and kinetics were calculated for the right leg only. All kinematic and kinetic calculations were performed using Visual3D software (C-motion Inc., Germantown, MD, USA) and analyzed using Matlab (R2020a, Mathworks, Natick, MA, USA).

### Experimental procedure

Participants’ weight was obtained with a digital scale. Height was measured with a tape measure attached to a wall. Weight and height were used to calculate the body mass index (BMI) in kg/m^2^. Waist circumference was measured at the level of the umbilicus (i.e., belly button) using a tape measure. We measured participants’ navicular height and the foot pressure distribution as participants stood on their right leg on the digital foot pressure mat.

We measured each participant’s preferred walking speed by averaging walking speed for a total of 20 trials from the gait carpet. Participants began each walking trial 2 meters before the edge of the carpet, and ended trial 2 meters after walking off of the carpet. They were instructed to walk at their normal pace (i.e., preferred walking speed). Following this, participants were positioned in the middle of the treadmill with one leg on each belt and asked to walk on the treadmill at their preferred speed (i.e., each participant’s walking speed measured using the gait carpet) and a standard speed (i.e., 1.25 m/s) for 2 minutes each.

### Statistical analysis

All statistical analyses were performed using IBM SPSS Statistics Ver.27 (IBM Corp.). Our initial analyses included descriptive statistics of participants’ demographic and clinical characteristics consisting of mean and standard deviations for our dependent variables. Pearson’s correlations were run to test associations between BMI and CSI; we confirmed the use of the CSI by testing the correlation between CSI and navicular height. The CSI (normal and lower) and BMI (normal and obese) were dichotomized and used to create four groups: adults with non-obese BMI scores (between 19 and 25 kg/m^2^) and normal arches (0.1–45.0%), non-obese BMI scores and lower arches (45.1–100%), obese BMI (greater than 30 kg/m^2^) and normal arches, and obese BMI and lower arches. Three-way mixed ANOVAs were run to examine the effects of BMI (non-obese vs obese), arch height (normal arch vs lower arch), and speed (participant’s preferred walking speed vs. standard pedestrian walking speed, 1.25 m/s) on spatiotemporal gait parameters (i.e., mean step length, step width, and double-limb support time), mean foot progression angle (i.e., toe-out angle), and peak angles and moments at ankle and knee joints (for the right leg only). Statistical significance was set at 0.05. Bonferroni corrections were used for post-hoc comparisons. Effect sizes were reported via partial eta squared (*ηp*^2^) after p-values, via small (0.01), medium (0.09), and large (0.25) effects.

## Results

Descriptive information for each group can be found in Table 1. BMI and CSI were significantly correlated (*r*(47) = 0.89, *p*<0.01). The partial correlation between CSI and navicular height controlling for BMI demonstrated a moderate association (*r*(47) = -0.68, *p*<0.01). Thus, a lower arch as indicated by a higher CSI was associated with a lower navicular height measure when controlling for BMI.

The three-way interaction between BMI, arch, and speed was not statistically significant for all outcome measures including spatiotemporal gait measures, foot progression angle, and lower-limb joint kinematics and kinetics (*ps*>0.05). There were no statistically significant two-way interactions between BMI and arch, between BMI and speed, and between arch and speed for all outcome measures (*ps*>0.05). Thus, only main effects are reported below.

### Spatiotemporal gait measures

There was a main effect of BMI on step length, step width, and double-limb support time, *F*(1,43) = 18.53, *p*<0.01, *ηp*^2^ = 0.30, *F*(1,43) = 6.83, *p*<0.01, *ηp*^2^ = 0.16, and *F*(1,43) = 26.65, *p*<0.01, *ηp*^2^ = 0.38, respectively (Table 2). Step length was shorter in individuals with obesity than individuals with normal weight (*p*<0.01). Step width and double-limb support time were greater in individuals with obesity than individuals with normal weight (*ps*<0.01). There were statistically significant effects of speed on step length and double-limb support time, *F*(1,43) = 39.56, *p*<0.01, *ηp*^2^ = 0.48 and *F*(1,43) = 46.81, *p*<0.01, *ηp*^2^ = 0.52, while no difference was found for step width (*p* = 0.29; Table 2). Individuals with obesity had shorter step lengths and longer double-limb support times at the preferred walking speed than standard walking speed (*ps*<0.01). No significant arch effect was found (*ps*>0.05).

**Table 2:** Means (standard deviations) for spatiotemporal gait parameters and foot progression angle during walking at each participant’s preferred walking speed (PWS) and pedestrian standard walking speed (SWS), 1.25 m/s, across BMI (non-obese and obese) and arch (normal arch and lower arch) groups. Statistical results using three-way mixed ANOVA are also shown (*P*_BMI_: *P* values of BMI effect; *P*_Arch_: *P* values of arch height effect; *P*_Speed_: *P* values of speed effect).

	Speed	Non-Obese		Obese		P values		
		Normal	Lower	Normal	Lower	*P*_BMI_	*P*_Arch_	*P*_Speed_
*Spatiotemporal gait measures*								
Step length (m)	PWS	0.51 (0.03)	0.53 (0.05)	0.44 (0.08)	0.46 (0.05)	<0.01**	0.15	<0.01**
	SWS	0.53 (0.03)	0.56 (0.03)	0.51 (0.04)	0.52 (0.04)			
Step width (m)	PWS	0.20 (0.03)	0.18 (0.02)	0.22 (0.02)	0.23 (0.05)	<0.01**	0.64	0.29
	SWS	0.20 (0.03)	0.18 (0.02)	0.21 (0.03)	0.22 (0.05)			
Double-limb support time (s)	PWS	0.11 (0.01)	0.11 (0.01)	0.15 (0.02)	0.14 (0.02)	<0.01**	0.33	<0.01**
	SWS	0.10 (0.01)	0.11 (0.01)	0.13 (0.01)	0.12 (0.01)			
*Foot progression angle (in degree)*								
Toe-out (−)/ Toe-in (+)	PWS	-3.54 (7.14)	1.34 (4.76)	-3.61 (4.62)	0.73 (4.85)	0.59	<0.01**	0.10
	SWS	-2.45 (6.55)	2.32 (3.35)	-3.60 (4.29)	0.83 (4.26)			

**P<0.01

*P<0.05.

### Foot progression angle

There was a statistically significant effect of arch on foot progression angle, *F*(1,43) = 9.06, *p*<0.01, *ηp*^2^ = 0.18, while no effects for BMI and speed were found (*ps*>0.05). At both preferred and standard walking speeds, the direction of the foot progression was significantly different between arch groups; the foot progression angle was positive (i.e., in-toeing) in the lower arch group versus negative (i.e., out-toeing) in the normal arch group (Table 2).

### Lower-limb joint kinematic and kinetic measures

**Fig 2:**
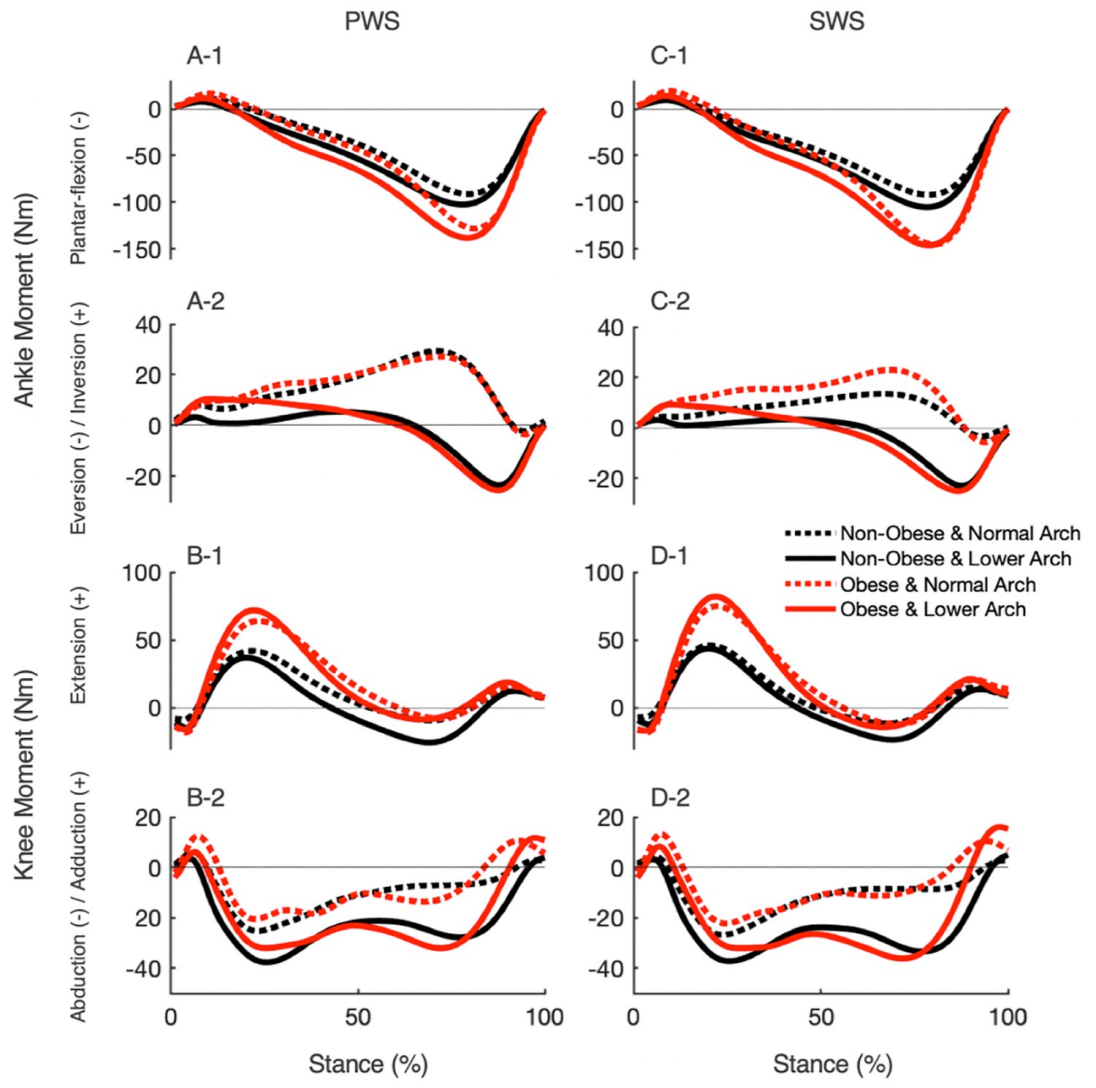
Group mean ankle and knee joint moments for the right leg. We plotted the internal ankle plantar-flexion moment (A-1, C-1), internal ankle eversion moment (A-2, C-2), internal knee extension moment (B-1, D-1), and internal knee abduction moment (B-2, D-2) over the stance phase of walking at preferred walking speed (PWS, A-B) and pedestrian standard walking speed, 1.25 m/s (SWS, C-D). Black lines represent the non-obese group, while red lines represent the obese group. Dashed lines represent the normal arch group, while solid lines represent the lower arch group.

**Fig 3:**
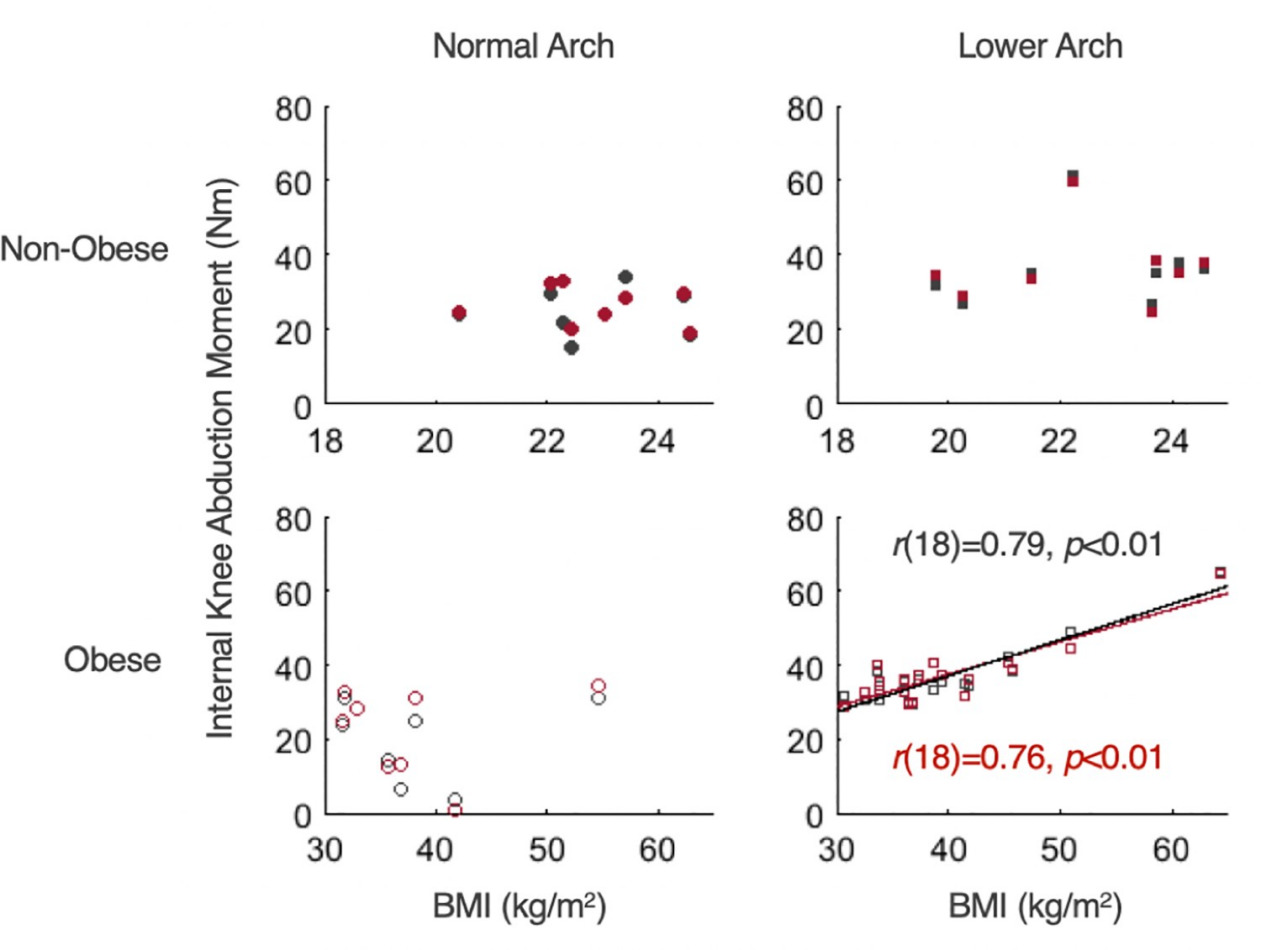
Relationship between BMI and peak internal knee abduction moment for four study groups. Pearson’s correlations (*r*) were significant for obese/lower arch group for both speeds, but not for non-obese/normal arch, non-obese/lower arch, and obese/normal arch groups.

For kinematic measures at the ankle and knee joints, there was a statistically significant effect of BMI on a knee adduction angle, *F*(1,43) = 38.18, *p*<0.01, *ηp*^2^ = 0.47, while all other simple main effects were not statistically significant (*ps*>0.05; Table 3). The knee adduction angle was greater in individuals with obesity compared to individuals with normal weight at both preferred and standard walking speeds (*p*<0.01). For kinetic measures at the ankle and knee joints, as shown in Fig 2 and Table 4, there were statistically significant main effects of BMI on peak internal ankle plantar-flexion moment and peak internal knee extension moment, *F*(1,43) = 28.55, *p*<0.01, *ηp*^2^ = 0.40 and *F*(1,43) = 18.76, *p*<0.01, *ηp*^2^ = 0.30, respectively. There were also significant effects of speed on the peak internal ankle plantar-flexion moment and the peak internal knee extension moment, *F*(1,43) = 17.23, *p*<0.01, *ηp*^2^ = 0.29 and *F*(1,43) = 20.37, *p*<0.01, *ηp*^2^ = 0.32, respectively. Peak internal ankle plantar-flexion and knee extension moments were less at the preferred walking speed than the standard walking speed (*p*<0.01). Individuals with obesity had greater peak internal ankle plantar-flexion and knee extension moments than individuals with normal weight (*p*<0.01). There were significant main effects of arch height on the peak internal ankle eversion moment, *F*(1,43) = 132.09, *p*<0.01, *ηp*^2^ = 0.75) and abduction moment, *F*(1,43) = 9.38, *p*<0.01, *ηp*^2^ = 0.18, and the first peak internal knee abduction moment, *F*(1,43) = 19.98, *p*<0.01, *ηp*^2^ = 0.32 (Fig 2 and Table 4). Peak internal ankle eversion and abduction, and knee abduction moments were greater in individuals with lower arches than individuals with normal arches (*ps*<0.01). For individuals with obesity and lower arches, as shown in Fig 3, there was a positive correlation between BMI and first peak internal knee abduction moment on both speeds (*r*(18) = 0.79, *p*<0.01 for preferred walking speed; *r*(18) = 0.76, *p*<0.01 for standard walking speed), while no correlation existed in the other groups (*p*>0.05).

**Table 3:** Means (standard deviations) for lower-limb joint angles during walking at each participant’s preferred walking speed (PWS) and pedestrian standard walking speed (SWS), 1.25 m/s, across BMI (non-obese and obese) and arch (normal arch and lower arch) groups. Statistical results using three-way mixed ANOVA are also shown (*P*_BMI_: *P* values of BMI effect; *P*_Arch_: *P* values of arch height effect; *P*_Speed_: *P* values of speed effect).

	Speed	Non-Obese		Obese		P values		
		Normal	Lower	Normal	Lower	*P*_BMI_	*P*_Arch_	*P*_Speed_
*Joint angle (in degree)*								
Ankle plantar-flexion	PWS	15.07 (6.35)	16.74 (6.24)	12.29 (3.28)	14.50 (3.42)	0.17	0.26	0.32
	SWS	15.93 (6.33)	16.71 (7.28)	13.31 (4.18)	14.69 (3.99)			
Ankle eversion	PWS	4.28 (2.59)	4.08 (1.45)	5.42 (2.79)	5.38 (2.47)	0.95	0.11	0.43
	SWS	4.04 (2.62)	4.84 (1.34)	4.97 (2.86)	5.06 (2.23)			
Ankle abduction	PWS	5.78 (4.18)	6.07 (2.43)	6.13 (3.50)	5.83 (4.31)	0.57	0.92	0.41
	SWS	5.85 (3.97)	6.58 (1.79)	6.01 (3.14)	5.81 (3.90)			
Knee flexion	PWS	41.89 (3.83)	39.16 (2.91)	41.92 (6.74)	38.67 (6.30)	0.83	0.07	0.13
	SWS	41.37 (4.39)	39.48 (2.89)	43.32 (5.53)	39.36 (6.42)			
Knee adduction	PWS	2.42 (2.63)	2.04 (2.95)	4.19 (4.61)	4.51 (3.92)	<0.01**	0.66	0.06
	SWS	1.84 (2.54)	1.74 (2.75)	4.01 (4.24)	5.05 (3.47)			
Knee internal rotation	PWS	3.29 (6.23)	4.38 (4.52)	5.43 (5.37)	5.88 (5.23)	0.09	0.25	0.49
	SWS	4.77 (6.58)	4.42 (6.56)	4.61 (7.56)	5.64 (5.56)			

**P<0.01

*P<0.05.

**Table 4:** Means (standard deviations) for lower-limb joint moments during walking at each participant’s preferred walking speed (PWS) and pedestrian standard walking speed (SWS), 1.25 m/s, across BMI (non-obese and obese) and arch (normal arch and lower arch) groups. Statistical results using three-way mixed ANOVA are also shown (*P*_BMI_: *P* values of BMI effect; *P*_Arch_: *P* values of arch height effect; *P*_Speed_: *P* values of speed effect).

	Speed	Non-Obese		Obese		P values		
		Normal	Lower	Normal	Lower	*P*_BMI_	*P*_Arch_	*P*_Speed_
*Peak internal joint moment (Nm)*								
Ankle plantar-flexion	PWS	91.15 (20.37)	102.42 (23.18)	128.20 (28.21)	138.24 (27.43)	<0.01**	0.26	<0.01**
	SWS	92.01 (21.91)	105.19 (22.37)	145.20 (28.41)	146.03 (31.51)			
Ankle eversion	PWS	2.20 (2.70)	23.62 (6.03)	3.53 (3.69)	25.69 (7.18)	0.28	<0.01**	0.49
	SWS	3.44 (2.81)	23.03 (4.16)	5.75 (4.48)	25.14 (10.44)			
Ankle abduction	PWS	7.38 (2.45)	12.33 (3.05)	7.83 (3.55)	11.01 (4.94)	0.91	<0.01**	0.18
	SWS	7.32 (2.51)	10.53 (3.62)	7.91 (4.13)	11.33 (5.18)			
Knee extension	PWS	41.74 (14.85)	36.82 (24.93)	63.86 (23.99)	71.75 (26.23)	<0.01**	0.79	<0.01**
	SWS	45.92 (14.67)	43.60 (20.41)	74.84 (30.49)	81.98 (29.40)			
Knee abduction	PWS	25.32 (5.99)	37.69 (9.92)	20.49 (10.91)	32.06 (8.48)	0.29	<0.01**	0.08
	SWS	26.83 (5.77)	37.21 (9.17)	22.29 (11.99)	36.21 (10.54)			
Knee internal rotation	PWS	6.37 (1.87)	9.95 (2.10)	8.75 (3.76)	11.19 (5.41)	0.06	0.05	0.43
	SWS	6.19 (2.18)	7.89 (1.95)	8.85 (3.54)	12.12 (7.63)			

**P<0.01

*P<0.05.

## Discussion

The results of this study suggest that BMI and arch height are each associated with particular measures of ankle and knee joint mechanics during walking in healthy young adults: (i) a higher BMI with greater internal ankle plantar-flexion moment and internal knee extension moment and (ii) a lower arch height with greater internal peak ankle eversion and abduction moments and internal knee abduction moment. However, there was no significant interaction between BMI and arch height; BMI did not influence the association between arch height and joint kinematic and kinetic measures, and arch height did not influence the association between BMI and joint kinematic and kinetic measures. These results remained constant at both participants’ preferred walking speeds and pedestrian standard walking speed (i.e., 1.25 m/s). We intentionally emphasized absolute joint moments instead of normalized values to better represent the actual loads placed on the ankle and knee joints. Our rationale is supported by the fact that our participants with obesity had over 66% greater body mass than normal-weight participants, and knee OA development has been associated with increased mechanical loads [40, 41].

Previous studies have shown that the internal knee abduction moment (i.e., external knee adduction moment) is greater in adults with obesity compared to adults without obesity [11, 15, 42]. These findings have been used as evidence that obesity may increase the risk of developing medial compartment osteoarthritis (OA) [30, 43, 44]. In the current study, we found significant increases in the internal knee abduction moment during walking in adults with lower arch heights compared to normal arch heights, but not in adults with obesity compared to normal weight. We also found in-toeing foot placement and greater internal ankle eversion and abduction moments, surrogate measures of foot overpronation, observed in only individuals with a lower arch, regardless of BMI status. These findings suggest that the combination of obesity and foot arch type may contribute to the risk of lower-limb musculoskeletal injury in individuals with obesity.

In individuals with obesity and lower arches, there was a positive relationship between BMI and internal knee abduction moment on both speeds (Fig 3). The relationships between obesity and lower arches suggests that excessive foot pronation due to both excessive body weight and lower arches may co-occur with frontal plane knee alignment. Obesity could cause a malalignment in the lower body, subsequent damage to the joints over time, and a have devastating impact on postural stability leading to increased fall risks [43–45]. Lower arch heights may be a major factor influencing altered frontal plane knee alignment angles by mediating the effect that obesity has on knee OA disease progression. In individuals with obesity, malalignment was related to internal knee abduction moment, suggesting that arch height mediates the relationship between BMI and internal knee abduction moment [31]. The relationship between the BMI and internal knee abduction moment in individuals with obesity and lower arches may also be indicative of changes preceding knee OA [45]. However, we still do not know whether medial knee-joint loads are greater in lower arched adults with obesity who show excessive foot pronation.

Obesity was also associated with increased internal ankle plantar-flexion and knee extension moments. Our findings that greater sagittal plane ankle and knee joint moments for obese BMI versus normal BMI individuals is consistent with the previous findings that obesity increases the vertical ground reaction force with changes in lower-limb sagittal plane kinematics and kinetics [6, 15]. This suggests that changes solely due to obesity are likely specific to the sagittal plane. The fact that those with obese BMI and normal arch heights were likely affected by changes in the sagittal plane ankle and knee joint moments may explain why no associations between internal ankle eversion moment and internal knee abduction moment were observed in this group.

Our findings showed that there were significant effects of walking speed on spatiotemporal gait parameters and frontal plane joint moments at the ankle and knee. Regardless of BMI classification, adults who walked at the standard speed increased peak loads across lower-limb joints. This suggests that altered gait mechanics may be influenced by walking speed, and compound the effect of obesity on gait. In fact, studies that examine gait kinematics and kinetics generally report greater forces and moments in obese BMI versus non-obese BMI individuals, suggesting greater joint loads that likely contribute to the development and progression of osteoarthritis [6, 12, 46]. Our addition of how arch height may play a role in gait and musculoskeletal conditions in this population moves us closer to understanding the possible relationship between obesity and the presence of knee OA.

Our results have practical implications for those with obesity who wish to engage in regular physical activity; our findings provide a better understanding of how arch height may affect gait mechanics based on BMI classification. In particular, our findings suggest that intervening on arch height may rectify lower-limb malalignment, reduce the risk of musculoskeletal injury, and facilitate an increase in physical activity through walking. For example, adults with obesity and lower arches may be more likely to respond to activity modification if provided with increased arch support (e.g., custom-designed insoles and orthotics for flat feet). Specifically, internal ankle eversion/abduction moments and internal knee abduction moment (i.e., external knee adduction moment) would be more appropriate for studying orthotic devices for fallen arches. In contrast, findings on internal ankle plantar-flexion moment and internal knee extension moment could be used to design weight loss programs involving walking at faster speeds. Promoting an increase in physical activity for moderately obese BMI individuals may prevent them from transitioning to having severely obese BMI. The current findings are congruent with research suggesting that it is critical to address factors that contribute to the risk of musculoskeletal injury in individuals with obesity and to support an increase in physical activity [46].

We acknowledge that the present study has limitations. First, we intentionally recruited participants without comorbidities such as osteoarthritis, plantar fasciitis, or cardiovascular disease. Thus, the generalizability of our study is limited by the fact that our participants may not be representative of those with obesity and additional conditions. Second, we did not capture non-weight bearing arch height in participants. This did not allow us to capture a measure of foot flexibility. However, the focus of the present study was on foot structure during weight-bearing activities. Future studies that use different methodologies (e.g., using both weight-bearing and non-weight-bearing measures of foot flexibility) are needed to investigate direct links between changes in joint kinematics and kinetics during walking mediated by arch height and musculoskeletal injury in obese BMI individuals. Last, we experienced challenges associated with placing markers over soft tissue in the approximate location of the anterior superior iliac spine (ASIS) and greater trochanter, which is common in this population [47–49]. Therefore, we did not report the results of hip kinematics and kinetics with the relationship between obesity and arch height. Future studies that use different methodologies (e.g., using dual-energy X-ray absorptiometry images or biplanar fluoroscopy) are needed to improve the accuracy of marker-based motion capture in obese BMI individuals. Despite these limitations, our results provide important information about the relationship between obesity, arch height, and gait.

## Conclusions

Our findings suggest that arch height affects obesity-related changes in gait mechanics. Foot structure, especially foot arch height, may be a valuable mediator for detecting changes in gait kinematics and kinetics in individuals with obesity. Understanding the relationship between obesity, foot anatomy, and gait mechanics can be used to design interventions aimed at decreasing the risk of musculoskeletal injury and increasing physical activity.
